# Optimizing plant transporter expression in Xenopus oocytes

**DOI:** 10.1186/1746-4811-9-48

**Published:** 2013-12-20

**Authors:** Huimin Feng, Xiudong Xia, Xiaorong Fan, Guohua Xu, Anthony J Miller

**Affiliations:** 1 State Key Laboratory of Crop Genetics and Germplasm Enhancement, College of Resources and Environmental Sciences, Nanjing Agricultural University, Nanjing 210095, PR China; 2 Department of Metabolic Biology, John Innes Centre, Norwich Research Park NR4 7UH, UK

**Keywords:** DNA optimization, *Xenopus* oocyte, Plant, Nitrate transporter, Uptake, Electrophysiology

## Abstract

**Background:**

Rapid improvements in DNA synthesis technology are revolutionizing gene cloning and the characterization of their encoded proteins. *Xenopus laevis* oocytes are a commonly used heterologous system for the expression and functional characterization of membrane proteins. For many plant proteins, particularly transporters, low levels of expression can limit functional activity in these cells making it difficult to characterize the protein. Improvements in synthetic DNA technology now make it quick, easy and relatively cheap to optimize the codon usage of plant cDNAs for *Xenopus.* We have tested if this optimization process can improve the functional activity of a two-component plant nitrate transporter assayed in oocytes.

**Results:**

We used the generally available software (http://www.kazusa.or.jp/codon/; http://genomes.urv.es/OPTIMIZER/) to predict a DNA sequence for the plant gene that is better suited for *Xenopus laevis*. Rice *OsNAR2.1* and *OsNRT2.3a* DNA optimized sequences were commercially synthesized for *Xenopus* expression. The template DNA was used to synthesize cRNA using a commercially available kit. Oocytes were injected with cRNA mixture of optimized and original *OsNAR2.1* and *OsNRT2.3a*. Oocytes injected with cRNA obtained from using the optimized DNA template could accumulate significantly more NO_3_^-^ than the original genes after 16 h incubation in 0.5 mM Na^15^NO_3_. Two-electrode voltage clamp analysis of the oocytes confirmed that the codon optimized template resulted in significantly larger currents when compared with the original rice cDNA.

**Conclusion:**

The functional activity of a rice high affinity nitrate transporter in oocytes was improved by DNA codon optimization of the genes. This methodology offers the prospect for improved expression and better subsequent functional characterization of plant proteins in the *Xenopus* oocyte system.

## Background

Heterologous expression systems are often used for the functional characterization of a gene. *Xenopus laevis* oocytes are widely used to express membrane proteins and channels. Over twenty years ago, the first plant membrane proteins were expressed in oocytes and these were a hexose transporter and a K^+^ channel [1,2]. Since then, many plant membrane proteins including carriers [3-5], channels [6-9] and aquaporins [10-13] have been successfully expressed in oocytes. Oocyte expression was used to demonstrate function for the first plant nitrate transporter (Chl1, AtNRT1.1 or AtNPF6.3) that was identified and later for many more family members [3,14-20]. Some of the plant NRT2 nitrate transporter family members require a second gene NAR2 for function and this requirement was demonstrated using oocyte expression [4,5,21-23]. The high affinity rice nitrate transporter, OsNRT2.3a needs a partner protein, OsNAR2.1 for function in oocytes [22,23].

Although all organisms generally share the same genetic code, each genus has evolved a slightly different pattern of codon usage. Heterologous protein expression in a foreign host may be diminished by factors such as biased codon usage, GC content and repeat sequences. To overcome these limitations, codon optimization can be used to enhance gene expression in various host cells. Heterologous synthetic genes with codon optimization showed increased expression levels in various organisms including *E. coli*[24,25], yeast [26] and mammalian cells [27,28]. For many plant transporters expressed in *Xenopus* oocytes, the low levels of expression can often limit the functional assay, making the detailed characterization of the protein difficult. In the past, it was speculated that differing codon bias may explain the very low levels of expression of some plant proteins in oocytes [29]. Improvements in DNA synthesis technology have enabled the technique to be used for cost-effective gene cloning. Commercial suppliers make it possible to obtain the synthetic DNA with codon optimization in just a few weeks. In this study, DNA of the rice genes *OsNAR2.1* and *OsNRT2.3a* were codon optimized and synthesized for oocyte expression. The cRNA of *OsNAR2.1* and *OsNRT2.3a* were then synthesized using a commercially available kit. We compared how this process may improve the functional activity of plant nitrate transporter proteins expressed in oocytes. The nitrate transport activity was assayed using ^15^N-enriched nitrate uptake and the two-electrode voltage clamp technique.

## Results and discussion

### Codon optimization of *OsNAR2.1* and *OsNRT2.3a*

**Figure 1:**
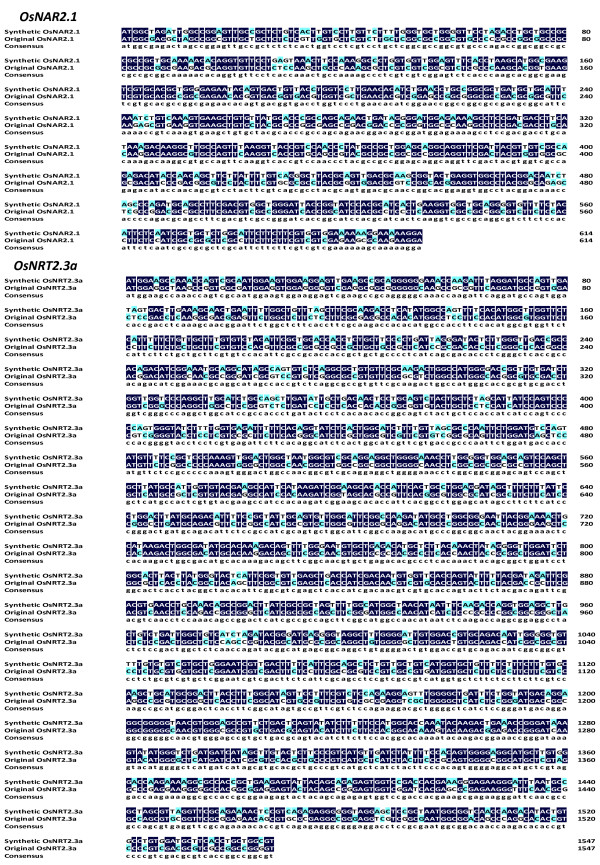
**Alignment of synthetic and original *OsNAR2.1 *and *OsNRT2.3a *open reading frame sequences highlighting the base changes.** In Table 1 more specific sequence differences are listed.

There are some general rules that emerge from the analysis of the preferred codons in Xenopus and these can be used to optimize a gene sequence for expression in oocytes [30]. Using the codon usage bias software for Xenopus (http://www.kazusa.or.jp/codon/; http://genomes.urv.es/OPTIMIZER/), the DNA gene sequence was optimized for *OsNAR2.1* (LOC_Os02g38230) and *OsNRT2.3a* (LOC_Os01g50820) and the resulting DNA sequences were synthesized by the Genescript Company and named syn-*OsNAR2.1 and syn-OsNRT2.3a*. After software optimization, the predicted GC content of syn-*OsNAR2.1* and syn-*OsNRT2.3a* was adjusted from 72.0 to 52.6% and 67.2 to 49.0% respectively, when compared with the original genes (Table 1). This change now makes the plant genes synthetic DNA much closer to the typical 50% GC content found in Xenopus [31]. For both synthetic DNAs the melting temperature (T_m_) was decreased and the number of repeat sequences was decreased in syn-*OsNAR2.1* (see Table 1). Sequence alignment of the open reading frames showed that syn-*OsNAR2.1* and syn-*OsNRT2.3a* shared 73% and 74% identity with the original genes (Figure 1), but the amino acid sequences did not change after optimization (see Additional file 1).

**Table 1:** **DNA sequence parameters of optimized plant transporter genes *OsNAR2.1 *** **and *OsNRT2.3a***

			***OsNAR2.1***		***OsNRT2.3a***	
			**Synthetic**	**Original**	**Synthetic**	**Original**
**GC content (%)**			52.6	71.0	49.0	67.2
**Repeat sequence**	Max direct repeat	Size:	8	11	10	13
		Distance:	7	177	984	993
		Frequency:	2	2	2	2
	Max inverted repeat	Size:	10	11	12	11
		Tm	27.3	55.7	48.8	53.6
		Start positions	3, 475	165, 54	230, 1497	764, 615
	Max dyad repeat	size	9	11	10	11
		Tm	27.5	59.8	31.9	50.2
		Start positions	151, 450	363, 540, 73	198, 1156	309, 1442
**Cis-acting element**	PolyT		0	0	0	0
	PolyA		0	0	0	0

### Nitrate uptake of oocytes

**Figure 2:**
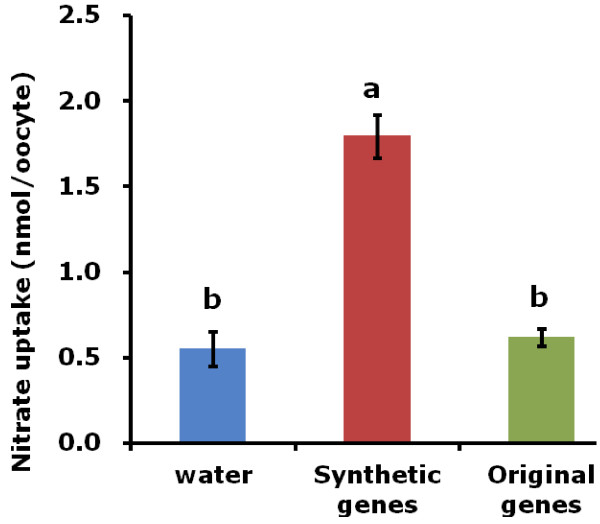
**Nitrate accumulation in Xenopus oocytes.** Mixed mRNA of either synthetic genes (synthetic *OsNAR2.1* and *OsNRT2.3a*) or the original genes (original *OsNAR2.1* and OsNRT2.3a) were injected into oocytes. Oocytes were incubated in MBS with 0.5 mM NaNO_3_ for 16 h and washed four times with NO_3_^-^ free MBS solution. Four oocytes were pooled for each sample. The values are means SE of four replicates with a and b indicating the statistical significance at p ≤ 0.05. The example shown is representative of the results from two frogs.

**Figure 3:**
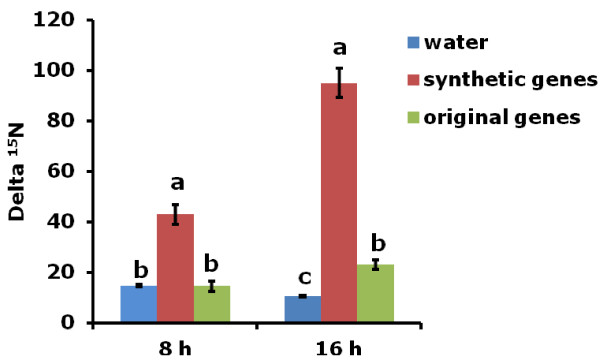
^**15**^**NO**_**3**_^**-**^**uptake in *Xenopus *oocytes.** Mixed mRNA of either synthetic genes (synthetic *OsNAR2.1* and *OsNRT2.3a*) or the original genes (original *OsNAR2.1* and OsNRT2.3a) were injected into oocytes. Single oocyte was incubated in MBS with 0.5 mM Na^15^NO_3_ for 8 h and 16 h, and then washed four times with cold 0.5 mM NaNO_3_ before ^15^N analysis. Data are averaged and SE of five oocytes. a, b and c indicate the significant difference at p ≤ 0.05 of same template DNA between different treatments at 5% of probability. The example shown is representative of the results from two frogs.

**Figure 4:**
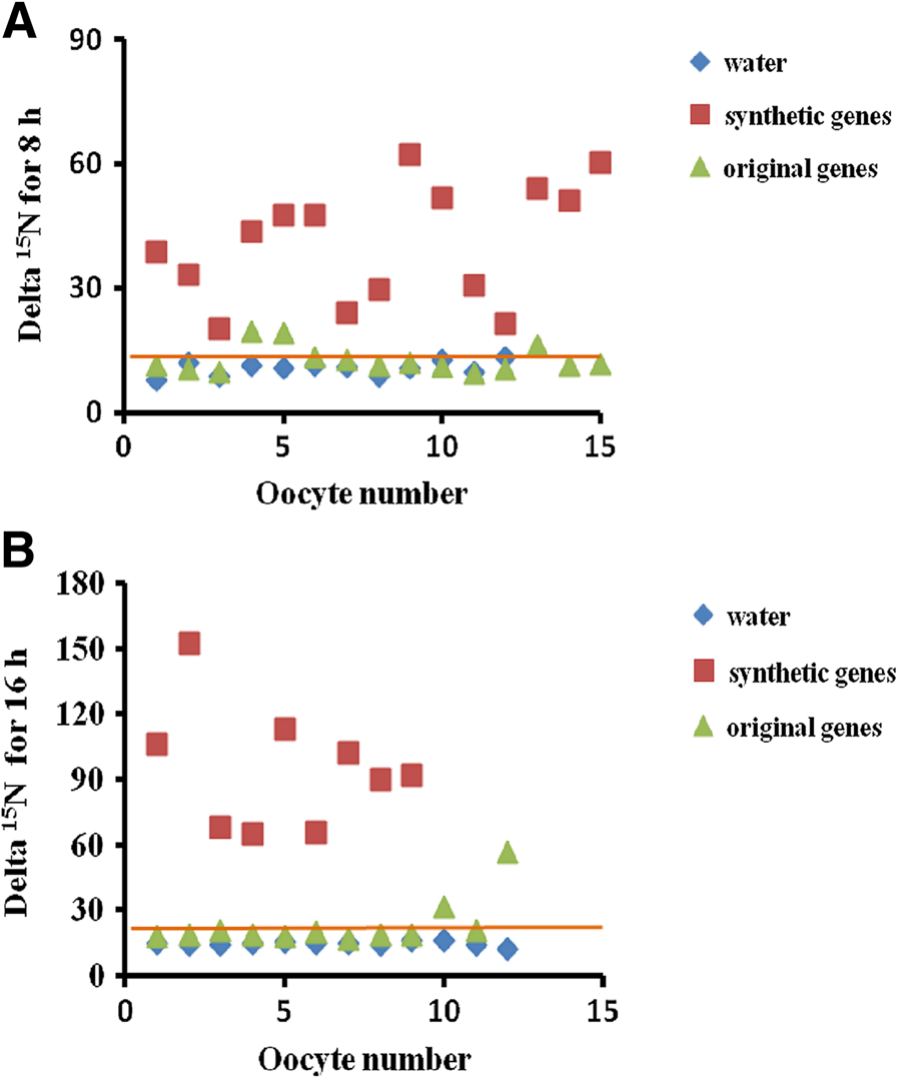
**Data spread analysis of**^**15**^**N-nitrate influx for individual oocytes injected with water or RNA.** Injected oocytes were incubated in MBS solution containing 0.5 mM Na^15^NO_3_ for 8 and 16 h. Delta ^15^N influx of individual oocytes injected with water (blue), RNA prepared from synthetic template DNA (red) and original genes (green) were compared. The data are from 10-15 cells after 8 h **(A)** or 16 h **(B)** incubation. The line (orange) represents the expected ^15^N influx value for an oocyte injected with the original plant DNA-template RNA. At both 8 h **(A)** and 16 h **(B)**, 100% of the synthetic-template RNA injected oocytes were above this threshold line, while the equivalent figure for the original plant DNA were 14 and 17% at 8 and 16 h respectively.

The original and synthetic (optimized) *OsNAR2.1* and *OsNRT2.3a* were subcloned in to expression vector pT7Ts [30] and then used as template to synthesize mRNA. Mixed mRNA of either synthetic genes (syn-*OsNAR2.1*:syn-*OsNRT2.3a*) or the original genes (ori-*OsNAR2.1*: ori-OsNRT2.3a) were injected into oocytes. Both RNA mixes were injected at the same ratio (*OsNAR2.1:*OsNRT2.3a, 25:50 ng). We used a colorimetric method to assay the amount of nitrate accumulated inside the oocyte. After 16 h incubation in MBS containing 0.5 mM NaNO_3_, oocytes injected with mRNA of synthetic genes showed increased NO_3_^-^ uptake when compared with the original genes (Figure 2). Similar results were obtained in 5 mM NaNO_3_ (Additional file 2)_._ These data did not show a significant difference between the original genes and water injected oocytes. In another set of experiments, injected oocytes were incubated in MBS containing 0.5 mM Na^15^NO_3_^-^ for 8 and 16 h. Compared to original genes, ^15^NO_3_^-^ uptake of single oocyte injected with synthetic genes were greatly enhanced after 8 h and 16 h incubation (Figure 3). Individual oocytes injected with synthetic gene mRNAs generally showed much greater ^15^NO_3_^-^ uptake after 8 h and 16 h when compared with oocytes injected with RNA made using the original plant DNA template (Figure 4A, B). The longer incubation time resulted in more nitrate accumulation also perhaps a greater concentration of transporter protein in the membrane has developed after 16 h. Statistical analysis showed that 100% of oocytes injected with synthetic DNA had significantly increased ^15^NO_3_^-^ uptake after 8 h and 16 h, while the equivalent figure was only 14% and 17% for the original plant template relative to water-injected controls (Figure 4). Presumably, the low percentage of nitrate transporter activity in oocytes injected with RNA from the plant DNA template can explain the results shown in Figure 2, where there was no significant difference between the total nitrate uptake of original genes-injected oocytes and water-injected ones in an 8 h uptake experiment.

### Electrophysiological analyses of oocytes

**Figure 5:**
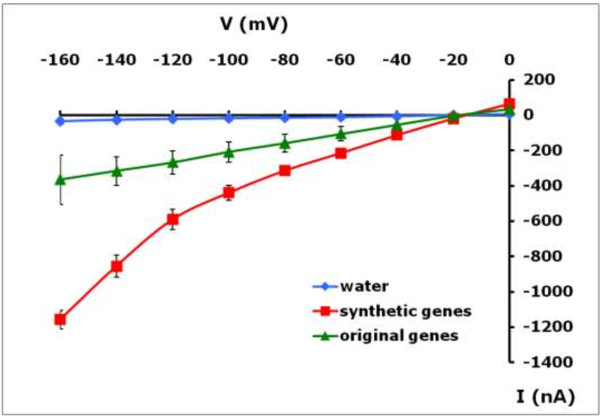
**Current–voltage difference curves for oocytes expressing the rice nitrate transporter OsNAR2.1/OsNRT2.3a.** These curves were recorded from oocytes injected with water (blue), synthetic (red) and original (green) genes. All the oocytes treated with 0.5 mM nitrate at pH 7.4. Results were average and SE values and obtained in three different oocytes from the same frog.

Two-electrode voltage clamp analysis was performed to record the voltage–current relationships of oocytes injected with mRNA [2,3]. After 48 h mRNA injected oocytes were treated with 0.5 mM NaNO_3_. Under these conditions when the plasma membrane voltage was clamped, in this example the nitrate-elicited currents of an oocyte injected with the synthetic genes was twice as large as an oocyte injected with RNA produced from the original plant DNA template (Figure 5). The electrophysiological measurements confirmed the accumulated nitrate (Figure 2) and ^15^N-nitrate influx (Figure 3) data in showing larger nitrate-elicited currents in oocytes injected with RNA made using the synthetic optimized DNA template. The ^15^N influx data was the average of 20–25 oocytes and showed an 8-fold advantage of using the optimized DNA. These data demonstrate the significant methodological advantage of using a template DNA that has been optimized for Xenopus expression.

### Enhanced transporter activity in oocytes

The expression of foreign proteins in oocytes has long been known to be improved by the inclusion of a polyA tail and the use of expression vectors that include Xenopus globin flanking UTR (untranslated region) sequence alongside the foreign DNA [1,29,30]. The polyA tail is recognized to improve mRNA stability and lifetime in the oocyte thereby improving the production of a foreign protein. The frog globin flanking UTR sequence is thought to make the heterologous cDNA more Xenopus-like and therefore thought to improve translation [29,30]. Similarly the mRNA produced from the synthetic DNA is more like Xenopus message and this has resulted in improved translational efficiency in the oocyte. For *OsNAR2.1* the removal of some repeat sequence (Table 1) may also have given better translation of the foreign protein. Making the GC content more like the 50% found in Xenopus [31] is likely to improve expression of plant genes. In Arabidopsis the GC content was reported as 44%, on the other hand in maize, rice and barley the figure was higher at >60% [32]. Mammals usually have around 44% GC in their coding sequence and experimental work directly comparing low-GC genes with their high-GC counterparts showed 100-fold greater expression in the GC-rich genes [33]. This study also showed that the mRNA degradation rate was independent of the GC content.

Together these data clearly show the methodological advantage for plant genes of using a synthetic template that has codon usage more like that found in Xenopus. It is widely accepted that optimized codons help to achieve faster translation rates and high accuracy in bacteria, yeast and mammalian cells [24-28,34] and we now show this has advantages for plant genes in Xenopus oocytes too.

## Conclusion

In this study, rice nitrate transporter *OsNAR2.1* and *OsNRT2.3a* were codon optimized for *Xenopus laevis*. Nitrate transport activity was analyzed and compared between oocytes injected with different sources of template DNA. The optimization changes the DNA, but not the protein sequence. Compared with the original plant genes, oocytes injected with optimized genes had increased nitrate uptake and larger currents in electrophysiological analyses suggesting that there was an increased level of protein expression. Taken together, these data show that the codon optimized template can give much improved expression and therefore provides a big advantage when aiming to functionally characterize a plant transporter protein in the Xenopus oocyte system. Although this may not be the case for all plant transporter genes the relatively cheap cost of DNA synthesis now makes this worthwhile when using oocyte expression.

## Methods

### Cloning and mRNA synthesis of *OsNAR2.1* and *OsNRT2.3a*

*OsNAR2.1* and *OsNRT2.3a* were codon optimized and synthesized by the Genescript Company. cDNAs were then subcloned into the BglII and SpeI sites of the oocyte expression vector pT7TS [35] using a directional cloning method. Original *OsNAR2.1* and *OsNRT2.3a* construct are as described previously [22]. Plasmid was linearized by BamH I (Roche) and purified by PCR purification kit (QIAGEN). The mRNA synthesis kit (mMESSAGE mMACHINE® T7 Kit, Ambion) was used to synthesized the mRNA of all genes. A compressed air system (Harvard) was used for injection [1]. Glass tips were calibrated for injection using known volumes and 1 μl mRNA mixtures (0.5 μg OsNAR2.1 and 1 μg OsNRT2.3a in total) were used to inject around 22–25 oocytes. Thus mRNA mixtures were injected as 25 ng: 50 ng, OsNAR2.1: OsNRT2.3a, and this weight ratio was chosen to reflect the differing molecular sizes and give similar molecular ratios [22,23]. Oocytes from three different frogs were used for the data shown.

### NO_3_^-^ accumulation and ^15^N-NO_3_^-^ uptake in oocytes

After injection, the oocytes were incubated for 2 days in NO_3_^-^ free MBS solution (88 mM NaCl, 1 mM KCl, 2.4 mM NaHCO_3_, 0.71 mM CaCl_2_, 0.82 mM MgSO_4_ and 15 mM HEPES, pH 7.4). The solution contains 10 g/ml sodium penicillin and 10 g/ml streptomycin sulphate. For NO_3_^-^ measurement, oocytes were incubated in MBS solution containing 0.5 mM NaNO_3_ at 18°C for 16 h. After incubation, oocytes were washed with NO_3_^-^ free MBS for four times. Four oocytes were collected as one sample in 1.5 mL tube, and 100 μl H_2_O was added into the tube. Lyses the oocytes and centrifuge the tube at 13000 rpm. The supernatants (40 μl) were collected for NO_3_^-^ assay using the kit (Nitrate/Nitrite Colorimetric Assay kit, Cayman).

For ^15^N-NO_3_^-^ measurement, oocytes were incubated in MBS solution containing 0.5 mM Na^15^NO_3_ with a 99% atom excess of ^15^N for 8 h and 16 h. oocytes were washed four times with ice-cold 0.5 mM NaNO_3_ MBS. Single oocyte was transferred to an empty tin capsule and then dried at 60°C for one week. Analysis for total ^15^N content using a continuous-flow isotope ratio mass spectrometer coupled with a carbon nitrogen elemental analyzer (ANCA-GSLMS; PDZ Europa). The delta-^15^N was calculated as described previously [4].

### Two-electrode voltage clamp analysis

The nitrate-elicited currents were recorded in oocyte using two-electrode voltage clamp method (pClamp 10.2, Axon). The oocytes were incubated in nitrate-free MBS and then treated with MBS containing 0.5 mM sodium nitrate. Membrane potential of oocytes was pulsed from 0 to -160 mV with 20 mV incremental steps. The currents were recorded to obtain current–voltage curves [2-4].

## Competing interests

The authors declare that they have no competing interests.

## Authors’ contributions

AJM and XF conceived the study. HF, XX and XF carried out the experiments. HF drafted the manuscript and all authors approved the final version.

## Supplementary Material

Additional file 1: Figure S1 Alignment of synthetic and original OsNAR2.1 and OsNRT2.3a amino acid sequences showing they encoded identical proteins. Click here for file

Additional file 2 **Nitrate accumulation in Xenopus oocytes in different concentration of nitrate.** Oocytes were incubated in MBS with 0.5 mM and 5 mM NaNO_3_ for 16 h and washed four times with NO_3_^-^ free MBS solution. Four oocytes were pooled for each sample. The values are means SE of four replicates with a and b indicating the statistical significance at p ≤ 0.05. Click here for file
