# Supplementary material for: Optimizing plant transporter expression in Xenopus oocytes
Source: Plant Methods. 2013 Dec 20;9:48. doi: 10.1186/1746-4811-9-48 (PMC3878178; doi:10.1186/1746-4811-9-48)
Supplement: Additional file 1: Figure S1 — Alignment of synthetic and original OsNAR2.1 and OsNRT2.3a amino acid sequences showing they encoded identical proteins. [file 1746-4811-9-48-S1.doc]

**Additional Figure1A comparing the amino acid sequence to show it is unchanged in synthetic DNAs.**
