# Supplementary figures and images for: Optimizing plant transporter expression in Xenopus oocytes
Source: Plant Methods. 2013 Dec 20;9:48. doi: 10.1186/1746-4811-9-48 (PMC3878178; doi:10.1186/1746-4811-9-48)

**Additional file 2**


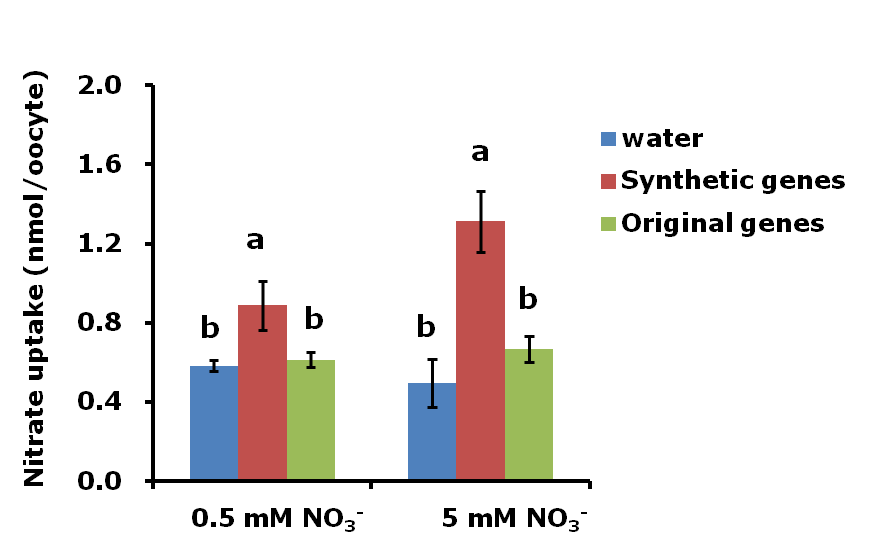

Supplement: Additional file 2 — Nitrate accumulation in Xenopus oocytes in different concentration of nitrate. Oocytes were incubated in MBS with 0.5 mM and 5 mM NaNO3 for 16 h and washed four times with NO3- free MBS solution. Four oocytes were pooled for each sample. The values are means SE of four replicates with a and b indicating the statistical significance at p ≤ 0.05. [file 1746-4811-9-48-S2.doc]
